# The ICE-AKI study: Impact analysis of a Clinical prediction rule and Electronic AKI alert in general medical patients

**DOI:** 10.1371/journal.pone.0200584

**Published:** 2018-08-08

**Authors:** Luke E. Hodgson, Paul J. Roderick, Richard M. Venn, Guiqing L. Yao, Borislav D. Dimitrov, Lui G. Forni

**Affiliations:** 1 Academic Unit of Primary Care and Population Sciences, Faculty of Medicine, University of Southampton, Southampton, Hampshire, United Kingdom; 2 Anaesthetics Department, Western Sussex Hospitals NHS Foundation Trust, Worthing Hospital, Worthing, West Sussex, United Kingdom; 3 Biostatistics Research Group, Department of Health Science, University of Leicester, Leicester, Leicestershire, United Kingdom; 4 Intensive Care Department, The Royal Surrey County Hospital NHS Foundation Trust, Guildford, Surrey, United Kingdom; 5 Department of Clinical & Experimental Medicine, University of Surrey, Guildford, Surrey, United Kingdom; University of Sao Paulo Medical School, BRAZIL

## Abstract

**Background:**

Acute kidney injury (AKI) is assoicated with high mortality and measures to improve risk stratification and early identification have been urgently called for. This study investigated whether an electronic clinical prediction rule (CPR) combined with an AKI e-alert could reduce hospital-acquired AKI (HA-AKI) and improve associated outcomes.

**Methods and findings:**

A controlled before-and-after study included 30,295 acute medical admissions to two adult non-specialist hospital sites in the South of England (two ten-month time periods, 2014–16); all included patients stayed at least one night and had at least two serum creatinine tests. In the second period at the intervention site a CPR flagged those at risk of AKI and an alert was generated for those with AKI; both alerts incorporated care bundles. Patients were followed-up until death or hospital discharge. Primary outcome was change in incident HA-AKI. Secondary outcomes in those developing HA-AKI included: in-hospital mortality, AKI progression and escalation of care. On difference-in-differences analysis incidence of HA-AKI reduced (odds ratio [OR] 0.990, 95% CI 0.981–1.000, P = 0.049). In-hospital mortality in HA-AKI cases reduced on difference-in-differences analysis (OR 0.924, 95% CI 0.858–0.996, P = 0.038) and unadjusted analysis (27.46% pre vs 21.67% post, OR 0.731, 95% CI 0.560–0.954, P = 0.021). Mortality in those flagged by the CPR significantly reduced (14% pre vs 11% post intervention, P = 0.008). Outcomes for community-acquired AKI (CA-AKI) cases did not change. A number of process measures significantly improved at the intervention site. Limitations include lack of randomization, and generalizability will require future investigation.

**Conclusions:**

In acute medical admissions a multi-modal intervention, including an electronically integrated CPR alongside an e-alert for those developing HA-AKI improved in-hospital outcomes. CA-AKI outcomes were not affected. The study provides a template for investigations utilising electronically generated prediction modelling. Further studies should assess generalisability and cost effectiveness.

**Trial registration:**

Clinicaltrials.org NCT03047382.

## Introduction

Acute kidney injury (AKI) is a clinical syndrome, defined through an acute increase in serum creatinine (SCr) and/or reduction in urine volume. However, there is a continuum of injury before sufficient loss of excretory kidney function can be detected with a change in SCr [1]. Up to 20% of adults admitted acutely to hospital meet AKI KDIGO (Kidney Disease: Improving Global Outcomes) criteria at, or during their stay, with high associated mortality [2]. This may reflect the limited ability of conventional markers to detect renal injury in a timely fashion, as well as heterogeneity in aetiology, which in turn provides some explanation as to why clinical trials of therapeutic interventions have proved disappointing [3].

There has been an international call to address deficits in recognition and management of patients with AKI, including early risk assessment at which point interventions could be most beneficial [1, 4, 5]. Implementation of clinical prediction rules (CPRs) is one strategy to improve risk assessment [6, 7], which relate multiple predictors, such as medical history and diagnostic tests, to risk of an outcome [8]. Unfortunately few CPRs have undergone impact analyses to investigate whether implementation is beneficial, with no such studies in the field of hospital-acquired AKI (HA-AKI). A second strategy, investigated predominantly by before-after observational studies, is the use of AKI e-alerts, with process measure improvements, such as reduced use of nephrotoxic medications, inconsistently reported [7]. Two studies from the UK reported reduced in-patient mortality and AKI progression in patients who had a linked care bundle completed [9, 10], and a large US before-after study suggested a significant reduction in mortality with the introduction of an AKI alert [11]. However, the only randomized controlled trial (RCT) reported to date utilising an e-alert showed no benefit [12]. An NHS patient safety alert in 2014 mandated English hospitals deploy an e-alert upon AKI development [13], however, uncertainty around the relative benefits of alerts and CPRs remains, necessitating ongoing rigorous evaluation [7, 14].

This impact analysis study employed one of the few available externally validated AKI CPRs–the Acute Kidney Injury Prediction Score (APS)—derived in a UK general medical population, which combines data on age, medical history and physiological parameters [15, 16]. The aims of the study included:

- In emergency medical admissions, without identifiable AKI at admission, could introduction of an electronically generated AKI CPR reduce the incidence of HA-AKI,
- In concert with the CPR, does an e-alert for new HA-AKI reduce associated complications including in-hospital mortality, escalation to intensive care and maximal AKI stage,
- Does identification of a group at high risk of developing HA-AKI improve outcomes,
- What is the effect of e-alerting community-acquired AKI (CA-AKI) at admission to hospital?

## Methods

Ethical approval was given by NHS Research Ethics Committee London—City Road and Hampstead (REC reference 13/LO/0960) and the study registered with clinicaltrials.org (NCT03047382) (S1 Protocol and S1 TREND Checklist available as supplementary files) [17]. A prospective controlled before-after impact analysis of the APS CPR and AKI e-alert was performed on two adult general acute medical units. Part of the same NHS Trust, the two sites were split into intervention and control and the study ran over two ten-month periods (2014–2016). There was a bed down period for the intervention of two months whilst the technology was safely introduced (Table A in S1 File for variables and weightings of the APS CPR). The Trust is a non-specialist hospital organisation of 870 beds, with the two sites 20 miles apart with similar catchment areas on the South Coast of England, though the control site has a higher rural population and less socio-economic deprivation [18]. Combined annual emergency department attendance is over 135,000 with 40–60 acute medical admissions per 24-hour period. The APS CPR was derived at the intervention site [15]. There was no cross-site contamination of clinical staff during the study: Consultant physicians and nursing staff do not work on both sites, nor do junior staff rotate between the hospitals (see Table 1 for demographic and clinical characteristics of the emergency admissions).

**Table 1:** Demographics and clinical characteristics of all patients pre and post intervention.

	Intervention Site			Control Site		
	Pre (n = 7,532)	Post (n = 8,636)	P value	Pre (n = 6,749)	Post (n = 7,378)	P value
**Age, mean (SD)**	75.1 (±16.9)	74.2 (±17.4)	0.001	74.6 (±16.7)	74.1 (±16.9)	0.149
**NEWS, median (IQR)**	1 (0–3)	1 (0–3)	0.009	2 (0–3)^#^	1 (0–3)	0.013
**Respiratory rate ≥20**	27.2% ^#^	25.8%^#^	0.054	20.2%	18.3%	0.003
**<Alert on AVPU scale**	1.3%	1.3%	0.944	1.2%	1.0%	0.339
**CKD**	47.7%	46.5%	0.122	49.1%	45.9%	<0.001
**Diabetes**	22.3%	23.7%	0.045	22.6%	23.2%	0.367
**Heart failure**	24.2%	24.4%	0.797	23.1%	23.4%	0.705
**Liver disease**	2.7%^#^	3.1%^#^	0.110	1.9%	2.0%	0.903
**Hypertension**	62.1%^#^	59.6%^#^	0.001	56.9%	55.1%	0.034
**Vascular disease**	10.3% ^#^	10.3%^#^	0.959	6.5%	7.6%	0.014

IQR–Interquartile range, SD–standard deviation. AVPU–best response: Alert, Vocal, Pain, Unresponsive, CKD–chronic kidney disease (baseline estimated glomerular filtration rate <60mls/min), NEWS–National early warning score, Respiratory rate–breaths/minute.

^#^ = significant difference (P<0.05) between sites during same period. T-test, Mann Whitney U tests or χ^2^.

At admission, all in-patients have physiological observations measured and entered via handheld systems into the clinical data software system (Patientrack Sydney, NSW, Australia). Previous ICD-10 coded history (heart failure, liver disease and diabetes mellitus), were retrieved and chronic kidney disease (CKD) was defined as an eGFR <60 mls/min/1.73m^2^ prior to admission (in those with an available baseline SCr); baseline SCr was defined using NHS England’s National algorithm [19]. KDIGO criteria for AKI was employed using only SCr as few of this patient group have routine urinary catheters placed (SCr increase of ≥1.5 from baseline value or absolute value ≥354μmol/l for CA-AKI; increase ≥1.5 from admission value or ≥26.5μmol/l within a rolling 48 hours during the first seven days of admission for HA-AKI). If no baseline was available, CA-AKI was assumed absent unless SCr was ≥354μmol/l. Patients were included if ≥18 years of age, stayed at least one night in the medical unit and had at least one SCr repeated.

Exclusion criteria:

- Patients moved directly from the A&E department to the ICU (as neither area uses the Patientrack data system),
- Aged <18 years,
- Non-medical (General Surgery, Trauma & Orthopaedics, Obstetrics and Gynaecology) admissions and,
- Discharged without spending a night in hospital.

**Fig 1:**
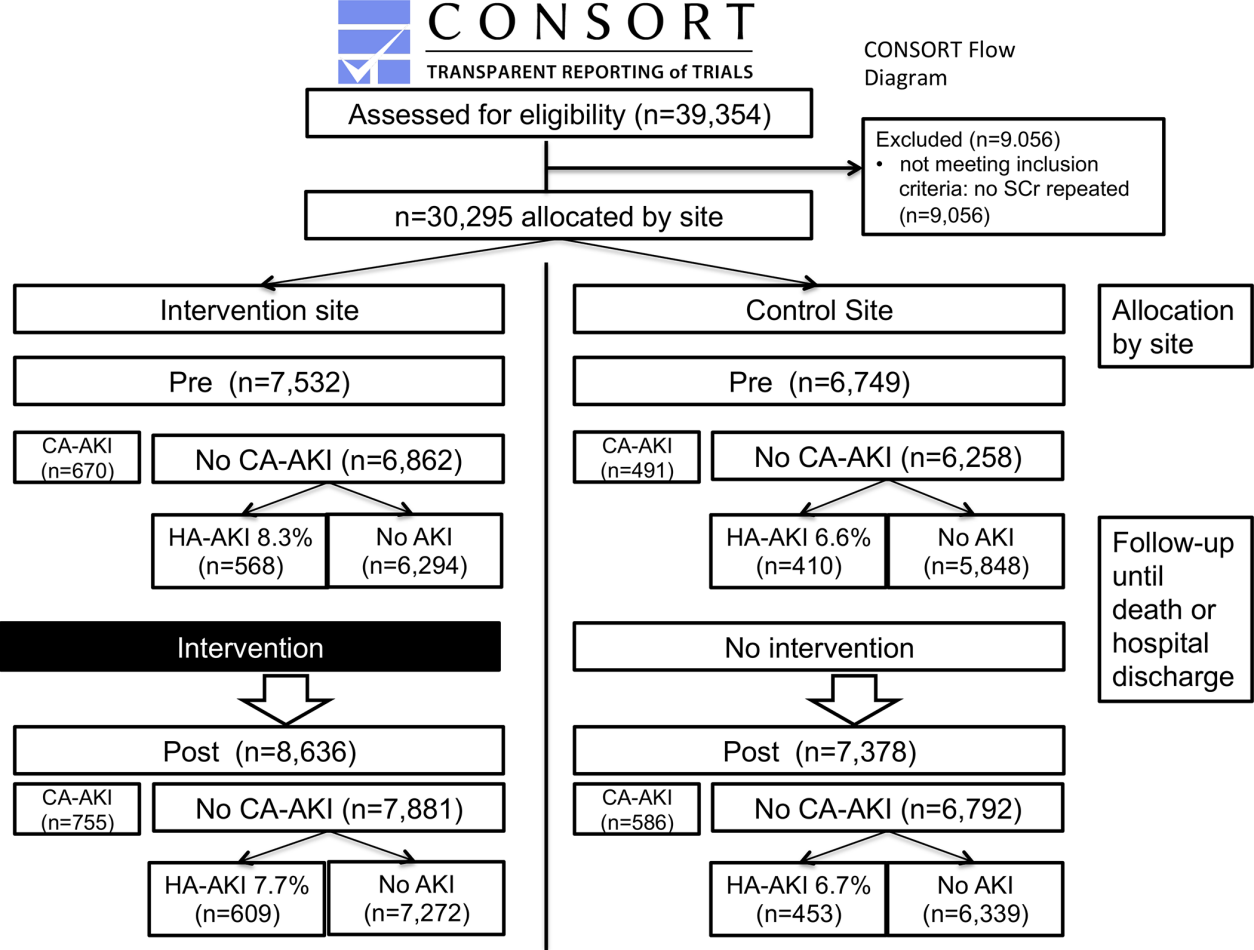
Consort diagram inclusion and exclusions. CA-AKI–Community-acquired AKI, HA-AKI–Hospital-acquired AKI, SCr–serum Creatinine.

Patients were followed-up until hospital discharge or in-hospital death (Fig 1 summarises the flow of participants in the study periods). As all patients admitted had an APS calculated automatically by the Patientrack system before any outcome had occurred, there were no missing demographic or physiological data at admission.

### Intervention

**Fig 2:**
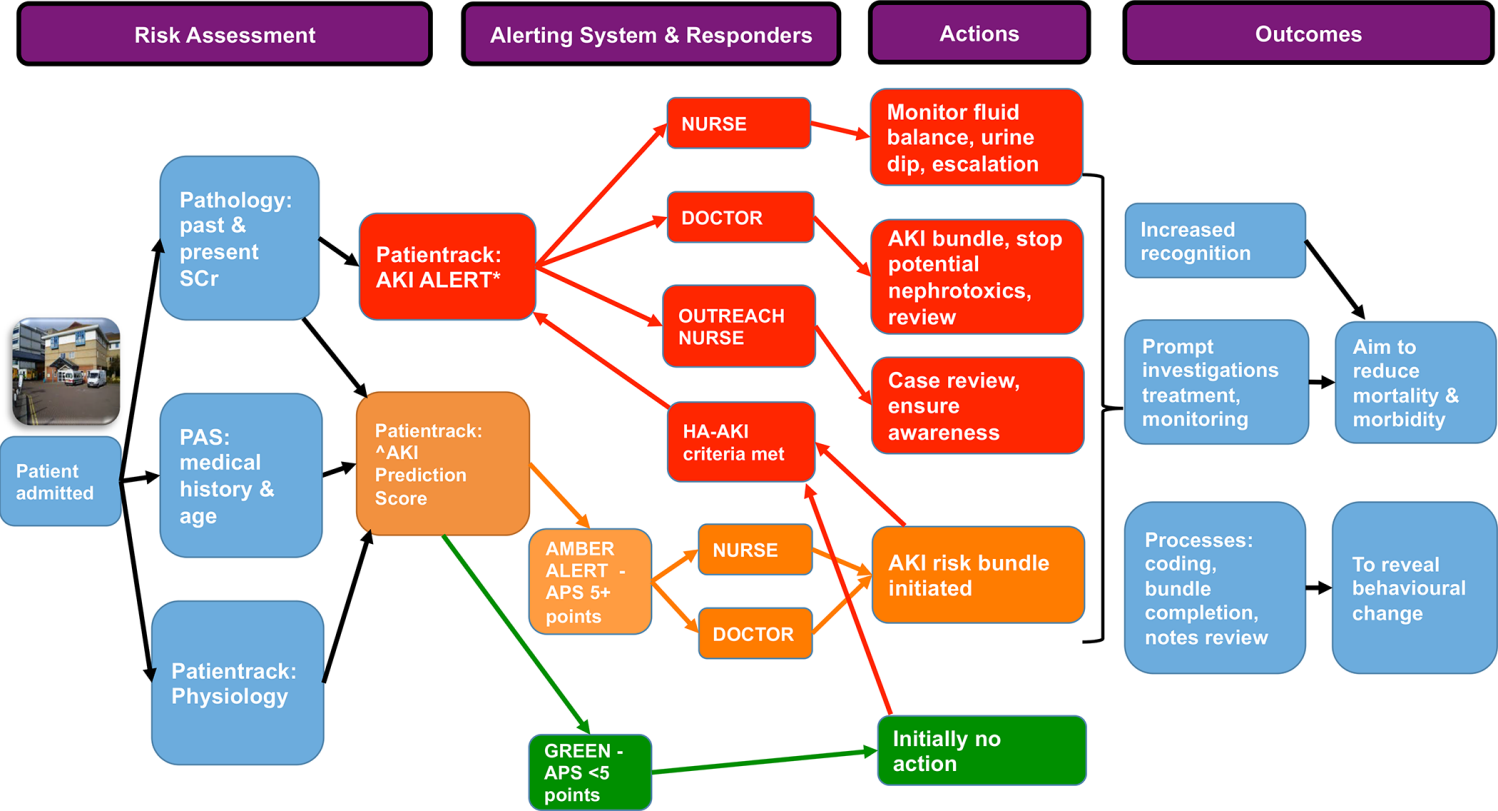
**Summary of intervention**. At the control site no alerts were generated. RED boxes = AKI (community or hospital-acquired), AMBER = APS ≥5 points–cut-off for flagging patient at risk of AKI, GREEN box–all other patients (APS <5 points). HA-AKI–Hospital-acquired AKI, PAS–Patient administration system, SCr–serum creatinine. Patientrack AKI ALERT* ^AKI Prediction Score (APS)–clinical prediction rule.

**Fig 3:**
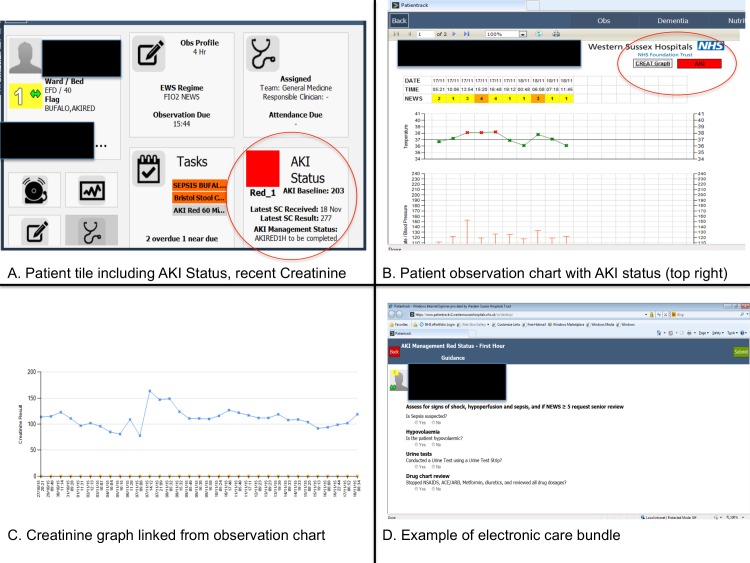
Top left (A): Patient tile indicating presence and stage of AKI, baseline SCr and care bundle task status; top right (B): electronic observation chart with AKI status (present or at risk) in top right of screen with link to SCr results; bottom left (C): graph of SCr; bottom right (D): AKI care bundle. SCr–serum creatinine. Note that details for illustration only and are not of a real patient.

The intervention is summarised in Fig 2. At admission those with an APS ≥5 points were flagged ‘at risk of AKI’ (coloured AMBER) in multiple electronic locations (Fig 3) and a care bundle generated (Tables B and C In S1 File). Those with an APS <5 points were classed GREEN, with no alert. This cut-off was chosen for its relatively high specificity and positive predictive value in derivation and validation, aiming to minimise false-positives that may limit uptake [15, 16]. Patients with CA-AKI were flagged RED, as were all who subsequently developed HA-AKI, with care bundles generated. Response was multidisciplinary: ward physicians, nurses, health care assistants and pharmacists, together with the critical care outreach nurse team reviewing patients remotely or at the bedside. None of the researchers involved in data analysis were involved in management of the patients and had access only to the fully anonymised individual level data and were blinded to other patient data, as well as components of the calculated APS score.

Barriers to integration and acceptance of CPRs and clinical decision support systems (CDSS) and expert guidance were considered prior to incorporation into the clinical workflow as part of a multi-faceted AKI guideline implementation [20, 21]. The controlled before-after design has been reported in previous impact analysis studies [22, 23]. The mandated NHS England patient safety alert limited the timeframe over which the study, a natural experiment, could be performed before the e-alert was introduced at the second (control) site.

### Outcomes

The primary outcome to assess the APS CPR was change in incidence of HA-AKI (KDIGO change in SCr) within seven days of hospital admission following intervention when adjusting for baseline differences between intervention and control sites. Other outcomes in those who developed HA-AKI included mortality in-hospital and seven days following admission, maximal increase in AKI KDIGO Stage and in SCr from admission, and ICU escalation. These outcomes were also assessed in patients highlighted by the CPR at high AKI risk (AMBER) and for patients with CA-AKI. Process measures were collected to attempt to explain the effects of the intervention. These included drug prescribing, completion of the suggested care bundles and a notes review of a randomly selected collection of HA-AKI cases.

### Statistical analysis

To avoid potential bias due to existing background differences between hospitals and temporal changes, we adopted the difference-in-differences approach by including time variable (before-after) and its interaction with the hospitals [24–29]. All models were adjusted by age and co-morbidities (ICD-10 diagnoses of heart failure, CKD, vascular disease, hypertension, diabetes mellitus and liver disease). The estimates of the intervention with a time period interaction term with associated 95% confidence intervals were reported. Difference-in-differences analysis makes two main assumptions [26]. First, the parallel trends assumption holds that intervention and control groups have equivalent trends for the outcome(s) prior to the start of the intervention. This implies that, without treatment, outcome(s) would be expected to change at the same rate. Secondly, the common shocks assumption infers that any event(s) occurring during or after the intervention will equally affect the intervention and control groups. To test whether the groups’ case mix did not change differentially over time, we examined for differences between patient characteristics before and after implementation in both groups (Table 1). Generalised estimating equations (GEE) with logistic link function were chosen to estimate the risks of binary outcomes between the intervention and control hospitals. GEE takes into account the clustering effects of multiple hospital admissions for the same individuals [30]. Generalised linear mixed models were employed to analyses continuous variables (change in SCr and hospital length of stay) including individual patients as a random effect [27]. Categorical outcomes were evaluated with the Cochran-Mantel-Haenszel χ^2^ tests. T-tests or Mann-Whitney U tests were used to compare continuous variables. Data were extracted fully anonymised on Microsoft^**®**^ Excel^**®**^ and all analyses were conducted using SAS (V9.4^®^).

## Results

Over the 20-month study period 30,295 admissions were analysed (Fig 1) with a mean age of 74.5 (standard deviation ±17.0), length of stay 11 days (±13.1) and in-hospital mortality 8.3%. AKI was present on admission (CA-AKI) in 8.3% (n = 2,502) and 7.3% (n = 2,040) developed HA-AKI. Table 1 shows the sites were relatively well matched at admission pre and post intervention, though the intervention site had higher lengths of stay, age, admission tachypnoea and some coded co-morbidities (liver disease, hypertension and vascular disease). Across all medical admissions between periods, at both sites there was no overall change in mortality, whilst length of stay reduced (Table D in S1 File). More patients were escalated to ICU at the intervention site in the second period (P = 0.013) though on difference-in-differences analysis there was no significant change in mortality or escalation to ICU.

### Incidence of HA-AKI

Difference-in-differences analysis suggested a reduction in HA-AKI at the intervention site (OR 0.990, 95% CI 0.981–1.000, P = 0.049). Unadjusted incidence of HA-AKI did not change significantly at the intervention (8.28% pre, 7.73% post intervention, OR 0.928, 95% CI 0.824–1.045, P = 0.223) or control sites (6.55% pre, 6.67% post intervention, OR 1.019, 95% CI 0.888–1.170, P = 0.805) (Table 2). In patients with only a single admission during the period of study, HA-AKI significantly reduced at the intervention site (9.4% pre vs 7.1% post intervention, OR 0.739, 95% CI 0.618–0.884, P = 0.001), with no change at the control site (6.7% pre, 6.2% post intervention, OR 0.910, 95% CI 0.735–1.127, P = 0.412).

**Table 2:** Incident HA-AKI pre and post intervention unadjusted and difference-in-differences analysis.

	Intervention site			Control site			Adjusted difference-in-differences change in outcome for intervention site vs control site
Outcome	Before (n = 6,862)	After (n = 7,881)	OR (95% CI), P value	Before (n = 6,258)	After (n = 6,792)	OR (95% CI), P value	OR (95% CI), P value
**HA-AKI**	8.28% (n = 568)	7.73% (n = 609)	0.928 (0.824–1.045), 0.223	6.55% (n = 410)	6.67% (n = 453)	1.019 (0.888–1.170), 0.805	0.990 (0.981–1.000), 0.049

HA-AKI–hospital-acquired AKI, OR–odds ratio.

### HA-AKI outcomes

**Fig 4:**
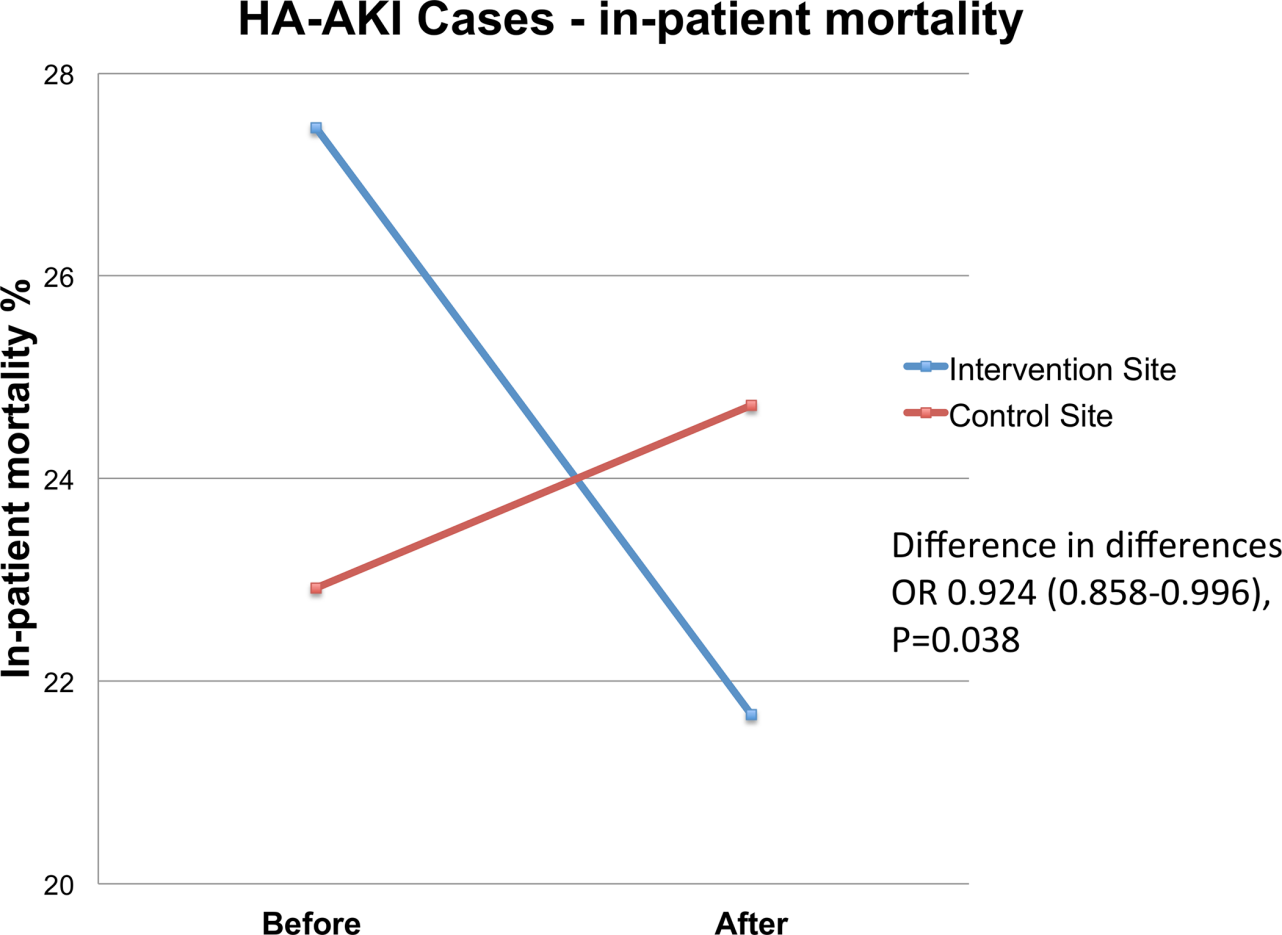
In-patient mortality in cases who developed HA-AKI before and after the intervention. HA-AKI–hospital-acquired AKI, OR–odds ratio (95% CI).

In cases of new HA-AKI in-patient mortality, using difference-in-differences analysis, significantly reduced at the intervention site (OR 0.924, 95% CI 0.858–0.996, P = 0.038) (Fig 4). Unadjusted in-patient mortality decreased at the intervention site (27.46% pre vs 21.67% post, OR 0.731, 95% CI 0.560–0.954, P = 0.021) with no change at control site (22.92% pre vs 24.72% post, OR 1.104, 95% CI 0.807–1.511, P = 0.576) (Table 3). A similar reduction in mortality by day seven was found. ICU escalation, progression to stage 3 AKI and maximal increase in SCr reduced at the intervention site, whilst increasing at the control site, without reaching statistical significance on difference-in-differences analysis. Length of stay did not change at either site.

**Table 3:** HA-AKI cases—pre and post intervention outcomes with adjusted difference-in-differences.

	Intervention Site			Control Site			Adjusted difference-in-differences change in outcome for intervention site vs control site
Outcome	Before (n = 568)	After (n = 609)	OR (95% CI), P value	Before (n = 410)	After (n = 453)	OR (95% CI), P value	OR (95% CI), P value
**In-patient** **mortality**	27.46% (n = 156)	21.67% (n = 132)	0.731 (0.560–0.954), 0.021	22.92% (n = 94)	24.72% (n = 112)	1.104 (0.807–1.511), 0.576	0.924 (0.858–0.996), 0.038
**7-day mortality**	16.20% (n = 92)	10.51% (n = 64)	0.608 (0.432–0.855), 0.004	9.02% (n = 37)	12.58% (n = 57)	1.451 (0.937–2.247), 0.101	0.907 (0.859–0.957), <0.001
**Stage 3 AKI**	8.27% (n = 47)	6.57% (n = 40)	0.779 (0.503–1.208), 0.268	4.15% (n = 17)	5.52% (n = 25)	1.350 (0.718–2.538), 0.429	P = 0.592
**ICU escalation**	7.39% (n = 42)	6.90% (n = 42)	0.928 (0.595–1.446), 0.821	7.07% (n = 29)	10.6% (n = 48)	1.557 (0.962–2.521), 0.074	0.980 (0.942–1.020), 0.319
**Peak SCr rise**	71.0 (±77.4)	66.1 (±76.3)	P = 0.269	64.6 (±61.0)	64.3 (±59.0)	P = 0.939	0.968 (0.931–1.007), 0.107
**Length of Stay**	14.7 (±15.4)	15.0 (±13.9)	P = 0.708	15.4 (±14.3)	13.7 (±12.9)	P = 0.064	P = 0.194

Mean (Standard deviation), HA-AKI–hospital-acquired AKI, ICU–intensive care unit, OR–odds ratio, SCr–serum creatinine, Stage 3 –KDIGO Staging x3 increase SCr.

At the intervention site, of those flagged as high risk (AMBER alert) 14% went on to develop HA-AKI vs 5% with no flag (GREEN), P<0.001. Following the intervention, in-patient mortality in this high-risk amber group significantly decreased (14% pre vs 11% post, OR 0.78, 0.66–0.94, P = 0.008) with no change at control site (Table 4 and Table E in S1 File). There were no significant changes in outcomes in those with CA-AKI at either site including mortality and AKI progression (Table 4 and Table E in S1 File). Process measure improvements at the intervention site vs control included an increased stopping of potentially nephrotoxic medications (P<0.001) and AKI documentation (P = 0.033) (Tables F and G in S1 File). Electronic bundle compliance (submission of a complete bundle) was 26% in those with AKI and 15% in all those flagged at risk.

**Table 4:** In-patient mortality in cases with CA-AKI and in those flagged at high-risk on admission by the CPR.

	Intervention Site			Control Site		
	Before	After	OR (95% CI), P-value	Before	After	OR (95% CI), P-value
CA-AKI Cases	n = 670	n = 755		n = 491	n = 586	OR (95% CI), P-value
In-patient mortality	23%	23%	1·01 (0·79–1·29), 0·95	19%	17%	0·86 (0·63–1·17), 0.34
AMBER (APS ≥5)	n = 2,057	n = 2,351		n = 1,810	n = 1,851	
In-patient Mortality	14%	11%	0·78 (0·66–0·94), 0·008	10%	10%	0·96 (0·78–1·20), 0·74

CA-AKI–community-acquired AKI, OR–odds ratio. AMBER—APS ≥5 points.

## Discussion

This is the first impact analysis study combining an AKI CPR and an AKI e-alert in hospitalised patients. The intervention was associated with a decrease in the incidence of new AKI but, importantly a significant improval in survival in those individuals who developed HA-AKI was also found. This may reflect a synergistic benefit of highlighting those at risk and de novo AKI, implying best practice would be institution of a care bundle in both groups, in tandem with a multimodal service improvement approach. Other outcome measures including escalation of care and progression of AKI were suggested. There was no change in outcome following introduction of an e-alert for patients with established AKI at admission to hospital, indicating the measures introduced did not affect the underlying disease process.

The primary aim of the study was to investigate whether an AKI CPR could prevent HA-AKI. On a difference-in-differences analysis a reduction was achieved. The treatment effect may have been limited due to a lack of specificity in the CPR—in derivation and validation the APS had moderate discrimination (AUROC of 0.65–0.72) and to avoid alert fatigue, only patients with a score ≥5 were alerted to [15, 16]. Furthermore, as AKI was only diagnosed through change in SCr, known to lag behind insult [31], it is possible a proportion classed as HA-AKI reflected community-acquired injury. Further important findings are that both in those highlighted as at high risk of developing AKI (AMBER) and in patients who subsequently developed HA-AKI, improved mortality outcomes were found, which may reflect systematic early recognition, with prompt care initiated even without documented evidence of a completed care bundle. Indeed without these alerts many of these patients would not have triggered an urgent response: for example median national early warning score (NEWS) was only 2 in the AMBER group, yet mortality ranged 11–14%. Based on the NEWS alone, such patients would not have been flagged high risk. This study provides evidence that application of best practice can translate into improved outcomes even in a heterogenous group at high risk of complications.

### Comparison with other studies

To date, the only RCT of an AKI e-alert showed no outcome benefit, though the intervention in that study was limited to an electronic link to practice guidelines [12]. In two UK studies, in patients who had developed AKI, a mortality benefit was reported in the 22–26% of those with a completed care bundle [9, 10]. A recent large before-after study in the US suggested a significant mortality benefit for the use of an AKI flag [11]. A before-after study where an alert was delivered the day after AKI recognition, found improved appropriate renal function monitoring, increased nephrology consultations and found less severe AKI after the intervention. However the groups had significant differences and there was no contemporaneous control.[32] One cluster RCT of a CDSS targeting patients with impaired renal found a significant increase in appropriate adjusting of prescribing which supports our finding of improved prescribing.[33] Though rarely reported, other CPR impact analysis studies have described successful integration into a CDSS [34, 35].

### Intervention uptake

The majority of studies reporting on the effects of alerts incorporated as part of CDSSs are medication related [36], however, such recommendations are often ignored [37–39]. Relatively little evidence explains why some succeed, with a paucity of evidence to demonstrate convincing improvement in patient outcomes [21, 40–42]. However, two systematic reviews [21, 43], suggested three independent factors influence success:

- automatic provision of decision support as part of clinician workflow at the time and location of decision-making,
- provision of recommendations rather than just assessments and,
- computer based decision support.

These factors and others were considered during study design, which provides a proof of concept for future investigations of complex healthcare interventions. Alerts could be quickly acknowledged, with a single alert at admission for those at risk, with a further alert on patients who met AKI criteria within the nursing clinical workflow [21, 44]. A cut-off on the APS CPR was chosen to minimise false positives to avoid blunting and eventual elimination of responses [45, 46]. Optimal visual field positioning and the use of appropriate colours (red and amber) were chosen as they are associated with an increase in hazard perception and prioritisation [47–50]. Despite these measures, compliance with electronic submission of care bundles was low ranging 15–26%, similar to previous AKI studies [9, 51, 52]. This could have been improved by making the alert interruptive however, this may have increased frustration and alert fatigue [9, 53, 54]. Physicians were expected to submit the completed bundle however it was not part of their workflow. However, crucially the intervention was multimodal: highly visible flagging in multiple electronic areas accessed by all members of the clinical team, alongside education, that may have changed group behavior not captured by a formal bundle submission. Whilst establishing conclusive proof of behavioural change is challenging, we found significant improvement in process measures, such as stopping potential nephrotoxic medication.

### Study strengths and weaknesses

This natural experiment introducing an electronic alert intervention at one site within a similar geographical area to a control site allows for unrelated change in disease occurrence and practice, with adjustment for confounders and has been described in previous impact studies [22, 23]. HA-AKI associated mortality at the two sites (20–26%) was similar to the other UK study showing a mortality benefit [9]. The process measure evaluation identified increased documentation, coding of AKI and stopping of nephrotoxic medications at the intervention site, which adds to the main data analysis suggesting both improved recognition and clinician behaviours following intervention (Tables F and G in S1 File).

The study took place as part of an information technology service improvement intervention that did not allow for a RCT design, with selection bias a concern. Between sites in both periods overall mortality was higher at the intervention site, as was the rate of HA-AKI, which may reflect differences in co-morbidities and socioeconomic factors. However, the before-after controlled design using difference-in-difference analyses is accepted as a way of detecting the effects of an intervention, and to control for confounders and secular trends [55, 56]. Outcomes did not improve in the control hospital suggesting other external factors did not significantly influence outcomes seen at the intervention site. However, given the difficulty of eliminating bias, a single study is unlikely to be definitive and replication and synthesis of evidence across studies is needed to support inferences about effectiveness [57]. Future studies of such organizational interventions could include a cluster RCT design to address for potential limitations of the presented study. A lack of power from two sites over ten-month periods could explain why outcomes, including ICU escalation and AKI severity, though improving at the intervention site, did not reach statistical significance–though for example in the second period significantly fewer patients with HA-AKI were escalated than at the control site. Generalisability may be limited by the study population on the South-Coast of England, being older (mean age 74) than the English NHS average admitted under general medicine (65 years), though the National average geriatric admission age is 78 [58]. Both sites are non-specialist general hospitals and the analysis used medical patients–future studies could examine generalisability to different hospital populations. However, our population probably reflects the majority of acute admissions in the UK given that the mean age of patients with CA-AKI in the RISK study being 75 years (unpublished, personal communication with study authors). The study did not assess whether impact was sustained or whether the intervention could be successfully introduced at the control site–the recommended fourth phase of impact analysis and implementation for example through a time-series design [20]. Calculation of the APS CPR past history relied on previous coded events attending hospital and could therefore have been incomplete. The predictive accuracy of the CPR could be improved by planned linkage with primary care as well as provide information of GP prescribed drugs, known to be associated with an increased risk of AKI.

### Future directions

**Fig 5:**
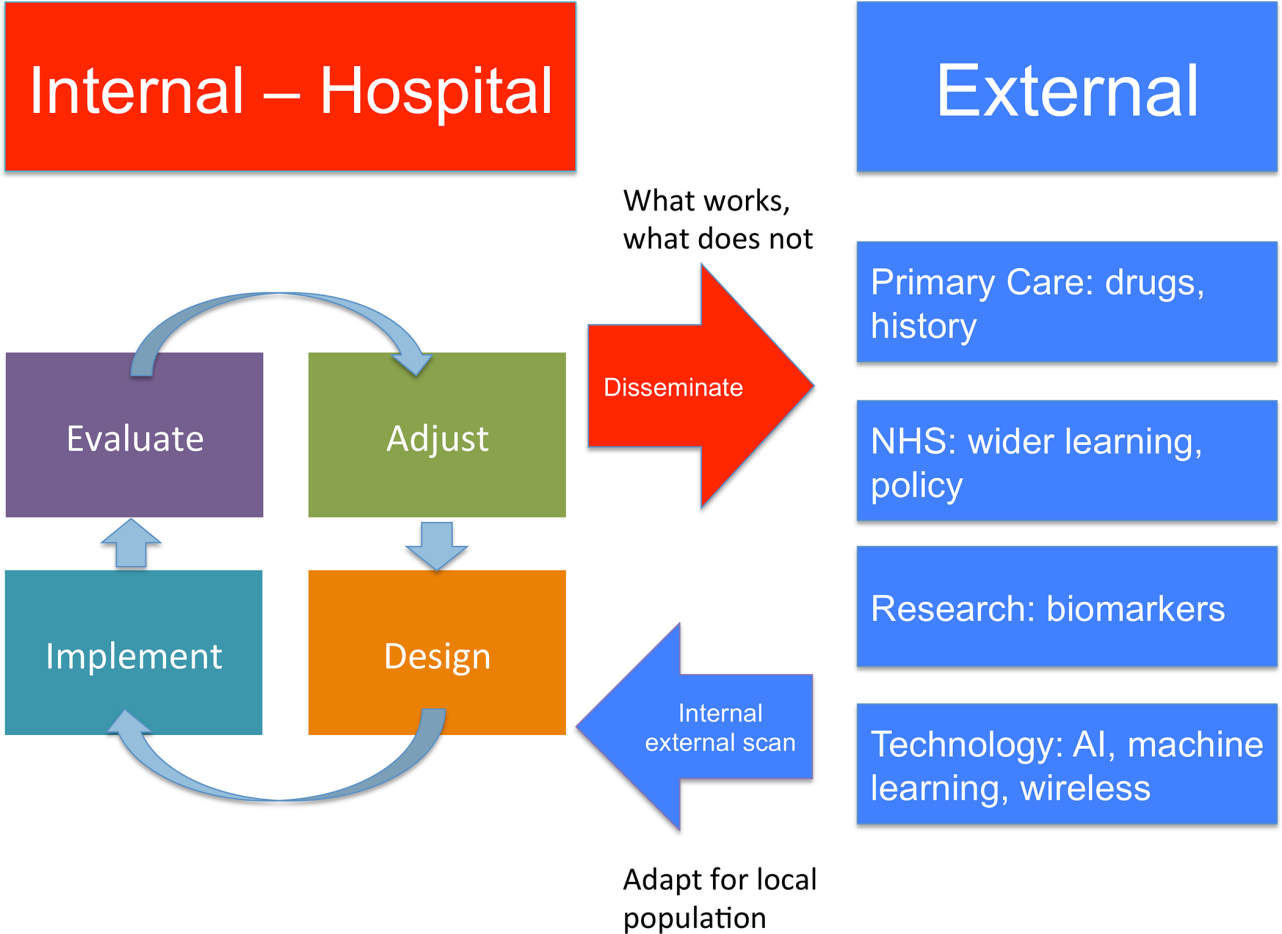
The learning health system–AKI as a case study. AI–artificial intelligence.

The concept of a continuously learning healthcare system was articulated by the Institute of Medicine in the US as a way of showing how evidence informs practice and vice versa in an iterative process of innovation and collaboration between research, information technology and clinical practice (summarized Fig 5) [59–61]. This study provides new insights in this emerging field, and future studies could build on knowledge for example by utilising more comprehensive records and dynamic data on physiological and blood parameters in the acute setting. This could enable the electronic record to display risk stratification at multiple time points from primary care to hospital admission with subsequent updating during an in-patient stay. Trends could provide powerful data overcoming limitations of current prediction models derived at a single time point. This study suggested a benefit from combining a CPR with an e-alert for HA-AKI. However, NHS England has required AKI alerting for patients presenting to hospital and this study does not provide evidence to support this to be sufficient to improve outcomes.

### Conclusions

In an impact analysis performed on general medical admissions, introduction of an AKI CPR was associated with a reduction in HA-AKI. In the context of a multi-faceted intervention, including an electronically generated care bundle for patients at risk of AKI using a CPR and e-alert for patients with HA-AKI, a significant reduction in mortality was demonstrated. An e-alert had no affect on outcomes for patients admitted with established AKI from the community. Further studies in the field are warranted to confirm or refute these findings and assess for generalisability to other hospital populations, healthcare systems, and for sustainability.

## Supporting information

S1 TREND Checklist (PDF)

S1 File (DOCX)

S1 Protocol (PDF)
