# Correction: The ICE-AKI study: Impact analysis of a Clinical prediction rule and Electronic AKI alert in general medical patients

**DOI:** 10.1371/journal.pone.0203183

**Published:** 2018-08-23

**Authors:** Luke E. Hodgson, Paul J. Roderick, Richard M. Venn, Guiqing L. Yao, Borislav D. Dimitrov, Lui G. Forni

## Notice of republication

An incorrect version of Fig 3 was published in error. This article was republished on August 17, 2018 to correct for this error. Please download this article again to view the correct version.
