# Supplementary material for: The ICE-AKI study: Impact analysis of a Clinical prediction rule and Electronic AKI alert in general medical patients
Source: PLoS One. 2018 Aug 8;13(8):e0200584. doi: 10.1371/journal.pone.0200584 (PMC6082509; doi:10.1371/journal.pone.0200584)
Supplement: S1 File — (DOCX) [file pone.0200584.s002.docx]

**S1 File**

Table A. Acute kidney injury prediction score (APS)

|  | **Points Scored** | | | |
| --- | --- | --- | --- | --- |
|  | **0** | **1** | **2** | **3** |
| Age (years) | **<60** |  | **60-79** | **≥80** |
| Respiratory Rate | **<20** | **≥20** |  |  |
| AVPU Score | **Alert** |  |  | **Other** |
| CKD Stage 3a-5 |  | **Y** |  |  |
| Heart failure |  | **Y** |  |  |
| Diabetes |  |  | **Y** |  |
| Liver disease |  |  |  | **Y** |

AVPU scale best response (alert, vocal, pain, unresponsive), CKD – chronic kidney disease (eGFR <60mls/min), Respiratory rates - breaths/minute.

| **AMBER** – at risk: advise to be reviewed/consider: | - Search for underlying cause: eg. If sepsis suspected send blood cultures - Assess fluid status & consider fluid bolus & re-assess, fluid balance chart, daily weights - Review drug chart & consider stopping potential nephrotoxics - Urine dip |
| --- | --- |
| **RED – AKI (Stage):** 1, 6 & 24 hour advice & tasks to be considered & submitted | Care bundle triggered immediately KDIGO criteria met for change in SCr >1.5 baseline at admission (or if >354μmol/L) or ≥26.5 μmol/l increase on rolling 48 hour period once admitted to hospital.  1 hour:   - Search for underlying cause of admission – eg. If sepsis suspected send blood cultures, IV antibiotics. - Fluid bolus & re-assess, fluid balance chart, daily weights - Review drug chart & stop potential nephrotoxics - Urine dip   6 hour:   - Re-review fluid status, consider escalation of care   24 hour:   - Where appropriate consider imaging renal tract - Consider other specific blood tests: autoimmune, CK. - If not imprivng for discussion with Nephrology |

Table B – Recommended actions for at risk (AMBER, ≥5 points on AKI CPR) and patients with AKI (RED). CPR – clinical prediction rule.

Table C. Differences between Intervention & Control sites post intervention

|  | **Intervention Site** | **Control Site** |
| --- | --- | --- |
| CA-AKI | - Red flag on Ward view, Observation chart & Patientrack reports | Red flag on Patientrack report |
|  | - Care bundle of actions | Usual Care |
|  | - Visible to Intensive Care Outreach team with aim to review <24hrs |  |
|  | - Pharmacy awareness |  |
|  | - Red flag automatically brought into e-discharge summary |  |
| Amber risk group | - Amber flag on Ward view, Observation chart & Patientrack reports |  |
|  | - Care bundle of actions |  |
|  | - Visible to Intensive Care Outreach team with review where appropriate |  |
|  | - Pharmacy awareness |  |
| HA-AKI | - Red flag on Ward view, Observation chart & Patientrack reports |  |
|  | - Care bundle of actions |  |
|  | - Visible to Intensive Care Outreach team with aim to review <24hrs |  |
|  | - Pharmacy awareness |  |
|  | - Red flag automatically brought into e-discharge summary |  |
| Education | - Mandatory training - E-learning | Mandatory training - E-learning |
|  | - Training & education on AKI & the CPR to junior doctors (lectures), Nurses, HCAs, Pharmacy (ward-based) |  |

AMBER - score of ≥5 on AKI prediction score (APS), CPR – clinical prediction rule, HCA - health care assistant.

Table D. All patients - pre and post intervention outcomes

|  | Intervention Site | | | Control Site | | |
| --- | --- | --- | --- | --- | --- | --- |
| **Metric** | Before (n=7532) | After (n=8636) | OR (95% CI), P value | Before (n=6749) | After (n=7378) | OR (95% CI), P value |
| **In-patient Mortality** | 9.82% (n=740) | 9.06% (n=782) | 0.914 (0.822-1.016), 0.100 | 7.13% (n=481) | 7.10% (n=524) | 0.996 (0.876-1.133), 0.974 |
| **7-day mortality** | 4.20% (n=316) | 3.61% (n=312) | 0.856 (0.730-1.004), 0.060 | 2.84% (n=192) | 2.71% (n=200) | 0.952 (0.778-1.163), 0.645 |
| **ICU escalation** | 2.46% (n=185) | 3.10% (n=268) | 1.272 (1.052-1.538), 0.013 | 2.44% (n=165) | 2.32% (n=171) | 0.947 (0.762-1.176), 0.659 |
| **Peak SCr rise** | 11.65 (±35.7) | 10.57 (±32.0) | P=0.045 | 9.05 (±27.6) | 9.22 (±27.4) | P=0.715 |
| **Length of stay** | 12.31 (±14.7) | 11.50 (±13.1) | P<0.001 | 10.69 (±13.4) | 9.57 (±10.9) | P<0.001 |

Mean (± standard deviation). ICU – intensive care unit, OR – odds ratio, SCr – serum creatinine.

| **Metric** | OR (95% CI), P value |
| --- | --- |
| **In-patient Mortality** | 0.995 (0.985-1.005), 0.328 |
| **7-day mortality** | 0.996 (0.989-1.003), 0.240 |
| **ICU escalation** | 1.003 (0.998-1.009), 0.255 |

Difference in differences estimates Intervention vs Control site, all patients.

|  | CA-AKI | | | | | | AMBER (APS ≥5) | | | | | | GREEN (APS <5) | | | | | |
| --- | --- | --- | --- | --- | --- | --- | --- | --- | --- | --- | --- | --- | --- | --- | --- | --- | --- | --- |
|  | Intervention Site | |  | Control Site | |  | Intervention Site | |  | Control Site | |  | Intervention Site | |  | Control Site | |  |
|  | Before (n=670) | After (n=755) | P-value, OR (95% CI) | Before (n=491) | After (n=586) | P-value, OR (95% CI) | Before (n=2,057) | After (n=2,351) | P-value, OR (95% CI) | Before (n=1,810) | After (n=1,851) | P-value, OR (95% CI) | Before (n=4,805) | After (n=5,533) | P-value, OR (95% CI) | Before (n=4,448) | After (n=4,941) | P-value, OR (95% CI) |
| In-patient mortality | 23% | 23%* | P=0·95, 1·01 (0·79-1·29) | 19% | 17% | P=0·34, 0·86 (0·63-1·17) | 14%* | 11%** | P=0·008, 0·78 (0·66-0·94) | 10% | 10% | P=0·742, 0·96 (0·78-1·20) | 6%* | 6%* | P=0·935, 0·99 (0·84-1·16) | 5% | 5% | P=0·436, 1·08 (0·89-1·31) |
| 7 day mortality | 12% | 12%* | P=0·87, 0·97 (0·71-1·34) | 9% | 7% | P=0·219, 0·75 (0·48-1·16) | 6%* | 4% | P=0·03 0·74 (0·56-0·97) | 4% | 4% | P=0·489 0·88 (0·63-1·24) | 3%* | 2% | P=0·437 0·90 (0·70-1·16) | 2% | 2% | P=0·432, 1·14 (0·83-1·56) |
| ICU escalation | 7% | 8% | P=0·92, 1·04 (0·07-1·54) | 8% | 7% | P=0·484, 0·85 (0·54-1·34) | 2% | 1% | P=0·08 0·67 (0·42-1·05) | 2% | 2% | P=1·0, 0·98 (0·59-1·63) | 2% | 3%* | P<0·001 1·68 (1·31-2·17) | 2% | 2% | P=0·719, 0·95 (0·71-1·26) |
| HA-AKI |  |  |  |  |  |  | 14% | 14% | P=1·0 0·97 (0·82-1·18) | 13% | 12% | P=0·446, 0·92 (0·76-1·12) | 6%* | 5% | P=0·103 0·87 (0·73-1·03) | 4% | 5% | P=0·122, 1·17 (0·96-1·43) |
| HA-AKI in-patient mortality |  |  |  |  |  |  | 31%* | 25% | P=0·067 0·71 (0·49-1·01) | 28% | 27% | P=1·0, 0·98 (0·65-1·49) | 24% | 19% | P=0·154, 0·74 (0·50-1·11) | 17% | 22% | P=0·178, 1·41 (0·86-2·31) |
| HA-AKI 7-day mortality |  |  |  |  |  |  | 18% | 11%** | P=0·020 0·57 (0·36-0·91) | 10% | 14% | P=0·113, 1·49 (0·84-2·64) | 14%* | 10% | P=0·096, 0·64 (0·39-1·07) | 8% | 11% | P=0·315, 1·49 (0·75-2·94) |
| HA-AKI ICU escalation |  |  |  |  |  |  | 9% | 4%** | P=0·027, 0·45 (0·23-0·91) | 4% | 8% | P=0·105, 2·01 (0·90-4·75) | 6% | 10% | P=0·127, 1·67 (0·90-3·07) | 11% | 13% | P=0·549, 1·23 (0·68-2·23) |
| Increase to AKI stage 3 | 5% | 5% | P=0·6, 0·86 (0·51-1·44) | 6% | 5% | P=0·486, 0·82 (0·47-1·41) | 9%* | 6% | P=0·267, 0·68 (0·36-1·26) | 3% | 5% | P=0·22, 1·98 (0·72-5·44) | 8% | 7% | P=0·756, 0·90 (0·49-1·66) | 6% | 6% | P=1·0, 0·98 (0·44-2·22) |
| Peak increase SCr | 0 (0-19) | 0 (0-11) | P=0·204 | 0 (0-10) | 0 (0-13) | P=0.744 | 51 (32-87) | 44 (29-74)** | P=0·026 | 46 (31-78) | 51 (34-78) | P=0·265 | 46 (30-72) | 45 (30-76) | P=0.981 | 47 (32-80) | 46 (31-72) | P=0.765 |

Table E(i) - Mortality, escalation and progression of AKI.

|  | CA-AKI | | | | | | AMBER (APS 5+) | | | | | | GREEN (APS <5) | | | | | |
| --- | --- | --- | --- | --- | --- | --- | --- | --- | --- | --- | --- | --- | --- | --- | --- | --- | --- | --- |
|  | **Intervention Site** | |  | **Control Site** | |  | **Intervention Site** | |  | **Control Site** | |  | **Intervention Site** | |  | **Control Site** | |  |
|  | **Before (n=670)** | **After (n=755)** | P-value, OR (95% CI) | **Before (n=491)** | **After (n=586)** | P-value, OR (95% CI) | **Before (n=2,057)** | **After (n=2,351)** | P-value, OR (95% CI) | **Before (n=1,810)** | **After (n=1,851)** | P-value, OR (95% CI) | **Before (n=4,805)** | **After (n=5,530)** | P-value, OR (95% CI) | **Before (n=4,448)** | **After (n=4,941)** | P-value, OR (95% CI) |
| **Age** | 80 (70-88) | 81 (70-87) | P=0.971 | 81 (70-87) | 80 (71-87) | P=0.848 | 85 (78-90) | 85 (78-90) | P=0.178 | 84 (78-89) | 84 (77-89) | P=0.312 | 76 (61-85)* | 72 (58-85)** | P=0.001 | 75 (61-84) | 75 (60-84) | P=0.620 |
| **LOS** | 9 (4-18) | 8 (4-17) | P=0.356 | 8 (3-16) | 8 (3-16) | P=0.823 | 9 (4-19)* | 9 (4-18)* | P=0.346 | 8 (4-17) | 8 (3-15)** | P=0.02 | 6 (3-15)* | 6 (2-14)** | P=0.012 | 5 (2-12) | 5 (2-11)** * | P=0.001 |
| **CCF** | 33% | 33% | P=0·866, 0·98 (0·79-1·22) | 32% | 33% | P=0·845, 1·03 (0·80-1·33) | 54% | 54% | P=0·954, 1·0 (0·89-1·13) | 52% | 54% | P=0·260, 1·08 (0·95-1·23) | 10% | 11% | P=0·50 1·05 (0·92-1·19) | 10% | 11% | P=0·417, 1·06 (0·93-1·21) |
| **DM** | 33% | 35%* | P=0·401, 1·10 (0·88-1·37) | 35% | 30%** | P=0·05, 0·77 (0·60-0·99) | 56% | 56% | P=0·692, 1·03 (0·91-1·16) | 57% | 60%* | P=0·094, 1·12 (0·98-1·28) | 7% | 8% | P=0·001 1·28 (1·10-1·48) | 7% | 9%** | P=0·004, 1·25 (1·07-1·45) |
| **LD** | 4% | 5%* | P=0·452, 1·125 (0·76-2·305) | 3% | 2% | P=0·430, 0·712 (0·33-1·56) | 6% | 7% | P=0·092, 1·24 (0·97-1·59) | 5% | 6% | P=0·196, 1·23 (0·91-1·67) | 1%* | 1%* | P=0·928 1·02 (0·72-1·45) | 1% | 1% | P=0·462, 0·82 (0·51-1·33) |
| **HTN** | 72% | 72%* | P=0·906, 1·02 (0·81-1·29) | 71% | 63%** | P=0·008, 0·71 (0·55-0·91) | 80% | 79% | P=0·43, 0·94 (0·81-1·09) | 79% | 78% | P=0·472, 0·94 (0·81-1·10) | 53%* | 49%* | P<0·001 0·87 (0·81-0·94) | 47% | 46% | P=0·443, 0·97 (0·89-1·05) |
| **Vascular** | 13% | 15% | P=0·492, 1·11 (0·82-1·50) | 10% | 12% | P=0·436, 1·18 (0·80-1·73) | 15%* | 16%* | P=0·528, 1·06 (0·90-1·25) | 11% | 12% | P=0·145, 1·16 (0·95-1·43) | 8%* | 8%* | P=0·394 0·94 (0·81-1·08) | 5% | 5%** | P=0·04, 1·22 (1·01-1·48) |
| **CKD** | 83% | 87% | P=0·102, 1·29 (0·96-1·72) | 85% | 87% | P=0·428, 1·15 (0·82-1·63) | 79% | 79% | P=0·768, 1·02 (0·89-1·18) | 83%* | 78%** | P=0·001, 0·75 (0·64-0·89) | 30% | 27% | P=0·01 0·89 (0·82-0·97) | 31%* | 29%** | P=0·007, 0·89 (0·81-0·97) |
| **NEWS** | 2 (1-4) | 2 (1-4) | P=0.022 | 2 (1-4) | 2 (1-4) | P=0.796 | 2 (1-4) | 2 (0-4)** | P=0.032 | 2 (1-4) | 2 (0-4)** | P=0.05 | 1 (0-3) | 1 (0-3) | P=0.342 | 1 (0-3) | 1 (0-3) | P=0.109 |
| **SBP <90** | 8% | 8% | P=1·0, 1·01 (0·68-1·49) | 13%* | 10% | P=0·250, 0·79 (0·54-1·15) | 3% | 3% | P=0·325, 0·83 (0·59-1·18) | 5%* | 4%* | P=0·285, 0·83 (0·60-1·15) | 2% | 2% | P=0·78 0·95 (0·73-1·26) | 3%* | 3%* | P=0·26, 0·87 (0·69-1·10) |
| **RR >20** | 32%* | 30%* | P=0·358, 0·90 (0·72-1·13) | 24% | 23% | P=0·666, 0·93 (0·71-1·24) | 46%* | 41%* ** | P=0·001, 0·81 (0·72-0·92) | 34% | 32% | P=0·277, 0·93 (0·81-1·06) | 19%* | 19%* | P=0·597 1·03 (0·93-1·13) | 14% | 12%** | P=0·015, 0·86 (0·76-0·97) |
| **<A on AVPU** | 3% | 2% | P=0·397, 0·74 (0·38-1·46) | 2% | 3% | P=0·686, 1·26 (0·56-2·84) | 3% | 3% | P=0·671, 1·08 (0·78-1·51) | 4% | 3% | P=0·52, 0·88 (0·62-1·27) | 0.30% | 0.20% | P=0·85 0·87 (0·40-1·87) | 0% | 0% | P=0·249, 0·45 (0·14-1·49) |

Table E(ii). Demographics, past medical history, physiological observations

Aps – AKI Prediction Score, Color code red = CA-AKI, Amber – APS ≥5 points flagged at risk of AKI, Green APS <5 points. SCr – serum creatinine. * = significant (P<0.05) difference between sites during the same period, ** = significant (P<0.05) difference between periods (pre vs post intervention) at the same site. Median (interquartile range). HA-AKI – hospital-acquired AKI, ICU – intensive care unit, LOS – length of stay, SCr – serum creatinine, Stage 3 – KDIGO staging x3 increase SCr.

Table F – process changes at the intervention site

| **Prescribing practice*** | - At the intervention site significantly more episodes of medications were stopped with AKI as the specific reason vs the control site (n=95 vs 36, P<0·001, OR 2·1 [1·41-3·10]. - Significantly more patients at the intervention site had ACEi/ARBs stopped vs control site (308 vs 146, P<0·001, OR 1·831 [1·493-2·247]) |
| --- | --- |
| **Bundle completion** | - For patients at risk of AKI on the APS (AMBER) and those with AKI a care bundle was recommended. Analysis was available for 7 of the 10 months following intervention with 15% of AMBER bundles (256/1,688 episodes) completed and 26% of bundles (258/983 episodes) completed for patients with AKI. |
| **Coding of AKI** | - ICD-10 coding of AKI increased at both sites with the intervention site having a larger increase (+21% vs +17%). - Coded AKI mortality at the intervention site significantly reduced (24% vs 20%, P=0·0057) with no significant change at the control site (20% vs 18% P=0·44). |
| **Notes review of processes documented** | - Random sample of patients who developed HA-AKI independently audited against recommended standards (documentation, investigations, management) - Significant increase in documentation of AKI in notes and on discharge and medication review at intervention site (see Table 6) |

*Introduction of electronic prescribing in the second period of the study (both sites), allowed for limited interrogation of prescribing behavior.

|  | **Intervention site** | | | | | **Control site** | | | |
| --- | --- | --- | --- | --- | --- | --- | --- | --- | --- |
|  | Pre | Post | P value | OR (95% CI) | Pre | | Post | P value | OR (95% CI) |
| **Documented AKI?** | 21% | 55% | **0·002** | **4·533 (1·763-11·657)** | 43% | | 28% | 0·177 | 0·524 (0·210-1·307) |
|  |  |  |  |  |  | |  |  |  |
| **Repeated U&Es** | 67% | 89% | **0·021** | **3·766 (1·218-11·640)** | 85% | | 72% | 0·189 | 0·456 (0·153-1·362) |
|  |  |  |  |  |  | |  |  |  |
| **Drugs checked documented** | 51% | 55% | 0·831 | 1·145 (0·493-2·660) | 25% | | 23% | 1·000 | 0·909 (0·312-2·645) |
|  |  |  |  |  |  | |  |  |  |
| **Fluid Plan documented** | 44% | 64% | 0·087 | 2·211 (0·935-5·224) | 50% | | 54% | 0·827 | 1·150 (0·486-2·723) |
|  |  |  |  |  |  | |  |  |  |
| **Search for cause documented** | 40% | 46% | 0·666 | 1·275 (0·544-2·988) | 55% | | 21% | 0·002 | 0·217 (0·083-0·567) |
|  |  |  |  |  |  | |  |  |  |
| **Appropriate special tests** | 34% | 37% | 0·822 | 1·143 (0·468-2·794) | 38% | | 16% | 0·041 | 0·319 (0·112-0·910) |
|  |  |  |  |  |  | |  |  |  |
| **AKI documented on discharge** | 20% | 42% | **0·049** | **2·857 (1·031-7·919)** | 27% | | 29% | 1·000 | 1·067 (0·369-3·080) |
|  |  |  |  |  |  | |  |  |  |

Table G(i) - Pre-Post intervention notes review.

Table G(ii) – Notes review comparison between sites, pre and post intervention.

|  | | **Pre intervention period** | | | | | **Post intervention period** | | | |
| --- | --- | --- | --- | --- | --- | --- | --- | --- | --- | --- |
|  | Intervention | | Control | P value | OR (95% CI) | **Intervention** | | **Control** | P value | OR (95% CI) |
| **Documented AKI?** | 21% | | 47% | **0·014** | **0·301 (0·118-0·764)** | 53% | | 30% | **0·033** | **2·710 (1·141-6·434)** |
|  |  | |  |  |  |  | |  |  |  |
| **Repeated U&Es** | 67% | | 85% | 0·076 | 0·366 (0·125-1·073) | 89% | | 72% | 0·062 | 3·019 (0·961-9·487) |
|  |  | |  |  |  |  | |  |  |  |
| **Drugs checked documented** | 51% | | 25% | **0·032** | **3·143 (1·157-8·534)** | 55% | | 23% | **0·004** | **3·960 (1·573-9·971)** |
|  |  | |  |  |  |  | |  |  |  |
| **Fluid Plan documented** | 44% | | 50% | 0·663 | 0·792 (0·334-1·878) | 64% | | 54% | 0·388 | 1·522 (0·645-3·589) |
|  |  | |  |  |  |  | |  |  |  |
| **Search for cause documented** | 40% | | 55% | 0·190 | 0·535 (0·224-1·280) | 46% | | 21% | **0·023** | **3·148 (1·224-8·095)** |
|  |  | |  |  |  |  | |  |  |  |
| **Appropriate special tests** | 34% | | 38% | 0·815 | 0·852 (0·337-2·151) | 37% | | 16% | **0·050** | **3·048 (1·100-8·441)** |
|  |  | |  |  |  |  | |  |  |  |
| **AKI documented on discharge** | 20% | | 27% | 0·580 | 0·667 (0·224-1·982) | 42% | | 29% | 0·322 | 1·786 (0·665-4·798) |
|  |  | |  |  |  |  | |  |  |  |

U&Es – urea and electrolytes, OR – odds ratio.
