# Supplementary material for: The ICE-AKI study: Impact analysis of a Clinical prediction rule and Electronic AKI alert in general medical patients
Source: PLoS One. 2018 Aug 8;13(8):e0200584. doi: 10.1371/journal.pone.0200584 (PMC6082509; doi:10.1371/journal.pone.0200584)
Supplement: S1 Protocol — (PDF) [file pone.0200584.s003.pdf]

SUSSEX NHS RESEARCH CONSORTIUM
CLINICAL TRIAL (NON-CTIMP)

PROTOCOL TITLE

'Preventable & Avoidable acute kidney injury (AKI) should never occur.' The use of an electronic prediction & identification alerting tool to deliver an individualised care bundle for hospital in-patients at risk of, or with confirmed AKI.

ESSENTIAL CONTACT INFORMATION

**Trial Manager name:**	Dr Lui Forni
**Trial Manager telephone:**	01903 285006
**Trial Manager email:**	Lui.Forni@wsht.nhs.uk

PROTOCOL IDENTIFICATION DETAILS

Version number: 3
Version date: 15/05/2013
Sponsor's ID number: 1539/WSHT/2013

CHIEF INVESTIGATOR

Name: Dr L Forni
Position: Consultant in Nephrology & Intensive Care
Institution: Western Sussex NHS Hospitals Trust
Address: Lyndhurst Road
Telephone: 01903 285006
Fax: 01903286786
Email: Lui.Forni@wsht.nhs.uk

SPONSOR

Institution: Sussex NHS Research Consortium
Address: Research Department, Worthing Hospital, Lyndhurst Road, Worthing BN11 2DH
Contact: Helen Evans
Position: Research governance manager
Telephone: 01903 285224
Fax: 01903 209884
Email: Helen.evans@wsht.nhs.uk

Name and details of Co-Investigator(s)

Name: Dr Richard Venn
Position: Consultant in Anaesthesia & Intensive Care Medicine
Institution: Western Sussex NHS Hospitals Trust
Address: Lyndhurst Rd
Telephone: 01903 285151
Fax: 01903286786
Email: Richard.Venn@wsht.nhs.uk
Roles: Joint Chief Investigator

Name: Luke Hodgson
Position: ITU Registrar
Institution: Western Sussex NHS Hospitals Trust
Address: Anaesthetics Department, Worthing Hospital, Lyndhurst Rd, Worthing
Telephone: 07734883040
Fax: 01903286786
Email: drlhodgson@gmail.com
Roles: Study Investigator

Name: Mr Simon Sturgeon
Position: Head of Informatics
Institution: Western Sussex NHS Hospitals Trust
Address: Department of Performance and Information, Lyndhurst Rd
Telephone:
Fax:
Email:
Roles: IT support

Name: Mr Mark Dennis
Position: Manager, Department of Performance and Information
Institution: Western Sussex NHS Hospitals Trust
Address: Department of Performance and Information, Lyndhurst Rd
Telephone:
Fax:
Email:
Roles: IT support

Name: Mr Andy Banks
Position: Information Manager
Institution: Western Sussex NHS Hospitals Trust
Address: Lyndhurst Rd
Telephone:
Fax:

Email:
Roles: IT support

Name: Outreach Nursing Team
Position:
Institution: Western Sussex NHS Hospitals Trust
Address: Lyndhurst Rd
Telephone:
Fax:
Email:
Roles: Support delivering care bundle

Name: Paul Roderick
Position: Professor Public health and statistics
Institution: Southampton Hospital
Address: Public Health Sciences and Medical Statistics, Level C, South Academic Block
Telephone: 02380796530
Fax: 02380796529
Email: pjr@soton.ac.uk
Roles: Statistics and Health Economics Support

Table of Contents

SUSSEX NHS RESEARCH CONSORTIUM 1
CLINICAL TRIAL (NON-CTIMP) 1
PROTOCOL TITLE 1
ESSENTIAL CONTACT INFORMATION 1
PROTOCOL IDENTIFICATION DETAILS 1
CHIEF INVESTIGATOR 2
SPONSOR 2
Name and details of Co-Investigator(s) 3
1. INTRODUCTION 8
1.1 BACKGROUND 8
2. STUDY OBJECTIVES 10
3. STUDY DESIGN 11
3.1 STUDY OUTCOME MEASURES 16
4. PARTICIPANT ENTRY 16
4.1 PRE-REGISTRATION EVALUATIONS 16
4.2 INCLUSION CRITERIA 16
4.3 EXCLUSION CRITERIA 16
4.4 WITHDRAWAL CRITERIA 16
5. ADVERSE EVENTS 17
6. ASSESSMENT AND FOLLOW-UP 17
7. STATISTICS AND DATA ANALYSIS 17
8. REGULATORY ISSUES 17
8.1 ETHICS APPROVAL 17
8.2 CONSENT 17
8.3 CONFIDENTIALITY 18
8.4 INDEMNITY 18
8.5 SPONSOR 18
8.6 FUNDING 18
8.7 AUDITS AND INSPECTIONS 18
9. STUDY MANAGEMENT 18
11. PUBLICATION POLICY 18
12. REFERENCES 19

GLOSSARY OF ABBREVIATIONS AND TECHNICAL TERMS

AKI	Acute Kidney Injury
APS	Acute Kidney injury Prediction Score
AVPU	level of consciousness – Alert/ responds to voice/Pain/Unresponsive
CCF	congestive cardiac failure
CKD	Chronic Kidney Disease
DM	Diabetes
EWS	Early warning system
eGFR	Estimated glomerular filtration rate – measure of kidney function
LOS	Length of stay
Patientrack	WSHT electronic observation system
PAS	Patient administration system
RR	Respiratory rate
Tachypnoea	Raised breaths
Tachycardia	Raised number of heart beats
WSHT	Western Sussex Hospitals NHS Trust

KEYWORDS
Acute Kidney injury, Alerting tool

STUDY SUMMARY

**TITLE**	**Electronic alerting tool to help prevent acute kidney injury**
**DESIGN**	Observational Cohort Study
**AIMS**	1. Externally validate an Acute Kidney injury Prediction Score (APS).<br>2. Identify patients who are at risk of developing Acute Kidney Injury following hospital admission to highlight the need for closer monitoring, allow preventative measures to be put in place and thus improve outcomes
**OUTCOME MEASURES**	• Incidence of Acute Kidney Injury (AKI)<br>• Magnitude of acute deterioration in Creatinine, eGFR<br>• Compliance with AKI checklist<br>• Mortality<br>• Morbidity – secondary complications including other organ failures<br>• Length of stay<br>• Requirement for renal replacement therapies<br>• Requirement for escalation to critical care
**POPULATION**	Patients admitted to WSHT Acute Medical Units & Becket Ward
**ELIGIBILITY**	All patients over the age of 18 admitted to the Acute medical Units and Becket Ward in WSHT
**DURATION**	24 months

1. INTRODUCTION

1.1 BACKGROUND

Acute kidney injury (AKI) is common in hospital in patients with a reported incidence of between 10 – 20% but can be as high as 70% in the critically ill.[1] Much of the early data examining the outcome of patients with AKI focused on the critically ill but increasingly, evidence is accruing which has highlighted the fact that patients with AKI regardless of clinical setting have worse outcomes.[2,3] Importantly, even small changes in renal function have been shown to be associated with significant increases in mortality. This is reflected not only in terms of mortality risk but also an increase in the development of chronic kidney disease with its associated complications.[4]

In 2009, The National Confidential Enquiry into Patient Outcome and Death (NCEPOD)[5] published its report “Adding insult to injury”, which examined the care of patients who had died in hospital with a primary diagnosis of acute kidney injury (AKI) or acute renal failure recorded on their death certificate. The overall findings were very critical of the care delivered to patients with renal impairment. Within the report, the group made some key recommendations regarding the admission and assessment for presence of AKI, the investigation of established AKI and its subsequent management. This particular study highlighted that in over 40% of cases there was deemed to be an unacceptable delay in diagnosing AKI and in 20% of cases the AKI was thought to be both predictable and avoidable. Parallels can be drawn with similar reports on the care of acute medical emergencies which highlighted the need for improved recognition of the deteriorating patient. This has lead to the use of track and trigger systems in tandem with a physiological warning score.

Several studies have tried to address this problem. In particular electronic systems have been studied where automatic alert warnings of patients with a rise in serum creatinine have been generated.[6,7] Such alert systems highlight a rise in creatinine after insult but do not inform as to the patients at risk of developing AKI following admission. The risk for development of AKI has been studied in specific patient groups predominantly in surgical and burns patients.[8,9] An AKI risk index for patients undergoing general surgery has been described where patients developing a rise in serum creatinine of over 177μmol/l (>2mg/dL) or AKI necessitating dialysis were found to have 11 independent preoperative predictors of AKI. In keeping with other work they also demonstrated an 8-fold increase in mortality in patients who developed AKI.[8] However, few attempts have been made to try to identify patients at risk of developing AKI when admitted to hospital as acute medical emergencies outside critical care. This is of particular relevance in that the majority of admissions to critical care with AKI are medical admissions. Attempts have been made to utilise electronic health records to produce a risk stratification model but this is not easily applied at the bedside and does not employ any physiological parameters.[10]

We have developed a simple, robust and practical scoring system utilising both physiological measurements, biochemical parameters and known co-morbidities that is easily calculable

and can identify patients at risk of developing AKI following hospital admission. We have named this the **A**cute Kidney injury **P**rediction **S**core (APS). Furthermore we have developed this into an automated electronic AKI prediction & identification tool utilising the hospital Patient Administration System (PAS), pathology database & Patientrack physiological Early Warning System (EWS) to deliver near real-time alerts to nursing and medical staff. The alert can trigger an automated e-mail to the patient's consultant and will advise on a care bundle which can be individualised for the patient.

Research Question/ Main Aims:

Phase 1 – is the APS externally valid? We will assess this by retrospective external validation of the score on the Chichester site.
Phase 2 - can the use of an electronic prediction & identification alerting tool to deliver an individualised care bundle for hospital in-patients at risk of or with confirmed AKI reduce:

1. The incidence of AKI in those patients 'at risk'
2. The harm (mortality, morbidity, Length of stay (LOS)) associated with failure to recognise those patients with confirmed AKI

Outcomes/ Analysis:

Phase 1 – external validation of the APS to predict the development of Acute Kidney Injury on another hospital site
Phase 2 – can the introduction of an alert impact on:

- Incidence of AKI
- Magnitude of acute deterioration in Creatinine and estimated glomerular filtration rate (eGFR)
- Mortality
- Morbidity – secondary complications including other organ failures
- LOS
- Requirement for renal replacement therapies
- Requirement for escalation to critical care

Main benefits & potential impact to NHS:

1. Health benefits immediate & projected medium to longer term
2. Cost-benefit analysis

The trial will be conducted in compliance with the principles of the Declaration of Helsinki 6th revision, the principles of ICH-GCP and all applicable regulatory requirements. The protocol is to be submitted for approval by a Research Ethics Committee.

1.2 RATIONALE FOR CURRENT STUDY

As the 2009 NCEPOD report highlighted, AKI is a common medical complication that is often diagnosed late and in many cases predictable and avoidable. From our own Worthing Hospital Audit data over six months through the Acute Medical Unit, AKI on admission to hospital had a prevalence of 11%. In addition, 7% of patients subsequently developed AKI during admission. Overall mortality for this combined group of patients with AKI was 19-20%.

Our innovative scoring system - the **A**cute Kidney injury **P**rediction **S**core (APS) - utilising physiological measurements, biochemical parameters and known co-morbidities can identify patients at risk of developing AKI following hospital admission. This can then deliver near real-time alerts to nursing and medical staff and will advise on a care bundle.

We would like to retrospectively externally validate the scoring system at another site to add methodological rigor. We will use two parallel interrupted time-series studies, on the Worthing & Chichester sites at WSHT, with segmented linear regression analysis in order to evaluate the longitudinal effect of intervention (use of the score and subsequent alert) and controls for trends in outcome.

2. STUDY OBJECTIVES

2.1 Trial objectives

1. Retrospectively externally validate the APS
2. Can the use of an electronic prediction & identification alerting tool to deliver an individualised care bundle for hospital in-patients at risk of or with confirmed AKI reduce:
    a. The incidence of AKI in those patients ‘at risk’
    b. The harm (mortality, morbidity, LOS) associated with failure to recognise those patients with confirmed AKI

2.2 Trial design

Initial retrospective external validation of the APS on the Chichester site.
2 parallel interrupted time-series design studies, on the Worthing & Chichester sites at WSHT, with segmented linear regression analysis in order to evaluate the longitudinal effect of intervention & controls for trends in outcome.

An in-patient episode will capture data from all these databases and this data will be transferred to an excel spreadsheet before anonymisation.

2.3 Trial statistics

Statistical methods

Sample size for original study

Worthing Audit data Feb 2010-July 2010: 3400 patients admitted through the Acute Medical Unit:

n= 1746 with a pre-admission creatinine (51.3%)
Of this cohort n=184 had AKI on admission (11%) 1561 (88%) did not
Of remaining group n=1219 did not develop AKI
N=95 (7%) developed AKI during admission

Figure 1: Patient Flow Chart

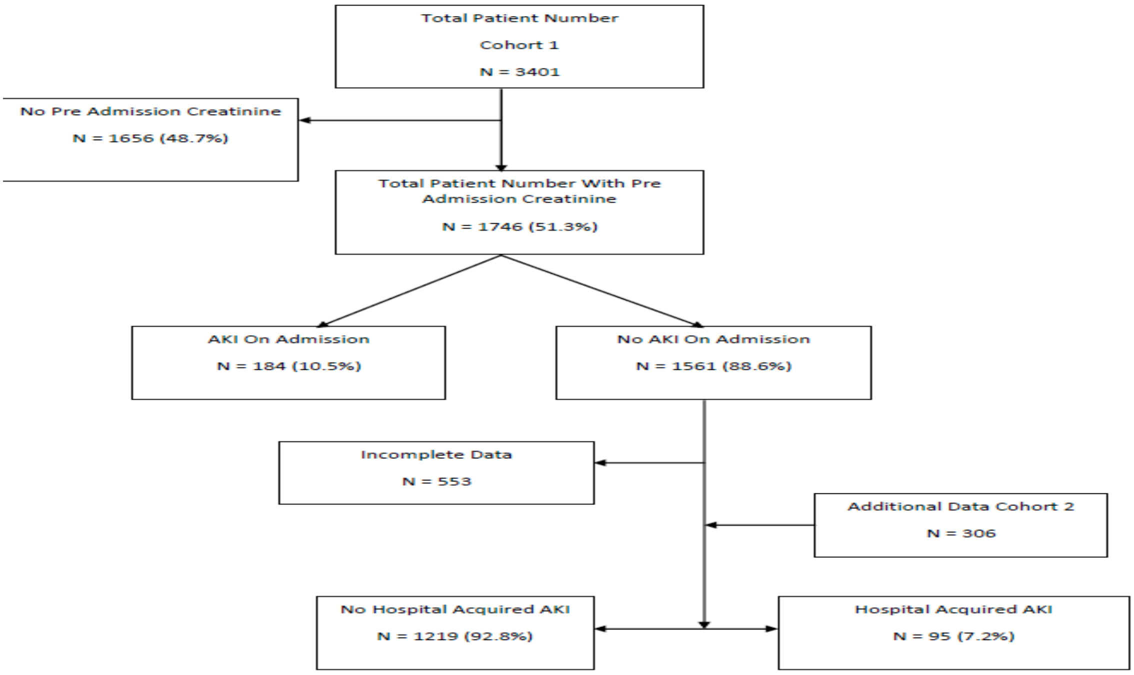

Figure 1 – flow chart for initial derivation of Acute kidney injury physiological scoring (APS) system. Based on 6 months data and a cohort of 1746 patients who had a recent creatinine result.

	Incidence	Hospital mortality
AKI on admission to hospital	11%	19%
Hospital acquired AKI	7%	20%

The initial retrospective validation on the St Richard's site will involve a similar number of patients to enable statistical significance in terms of incidence of acute kidney injury to be assessed. A similar number of patients will then be assessed prospectively once the Acute kidney injury alert is introduced onto the Worthing site to allow comparisons between the two sites.

3. STUDY DESIGN

2 parallel interrupted time-series design studies, on the Worthing & Chichester sites at WSHT, with segmented linear regression analysis in order to evaluate the longitudinal effect of intervention & controls for trends in outcome.

Initially (Phase 1) we will retrospectively review both sites to validate the score. Using the Chichester site this will add external validity to the APS score.

The alert will then be instituted (Phase 2) at the Worthing site during which time both sites will be monitored for the incidence of Acute kidney injury and adherence to the Patientrack Assessment Pack at the Worthing site. After interim analysis, the plan will then be to roll-out the alert to the Chichester site and further analyses will then be performed.
Worthing and Chichester are relatively close geographically and demographics are similar making the use of the Chichester site as the initial 'control' arm methodologically sound. If there are significant differences in the two populations in terms of AKI incidence and outcomes, they will be picked up in the initial Phase 1 part of the study

Duration: 24 months

Cohort: All medical patients admitted as emergencies to WSHT two sites Acute Medical Units (AMU) and Becket ward and spend at least one night as an in-patient.

Baseline data collection: data will be retrieved from the Patientrack, Pathology & PAS databases relevant to the 'AKI Patientrack Assessment Pack'. This data will be transferred to an excel spreadsheet and then anonymised. This data will be stored on the WSHT server & only hospital password protected computers will be able to access this data. Anonymised data for mathematical & statistical analysis will be transferred by password protected & encrypted memory sticks or secure NHS e-mail.

Introduction of 'AKI Patientrack Assessment Pack' to WSHT: every night PAS will be searched for relevant AKI score diagnosis on every new admission within the last 24hrs & this will be fed into the 'AKI' algorithm. Biochemistry will similarly be searched for the worst eGFR in the last 6/12 to identify the diagnosis Chronic kidney disease (CKD) for the AKI score. RR & AVPU will be fed directly from Patientrack into the algorithm. Therefore we will be able to identify those patients 'at risk of AKI' who have been admitted within the last 24 hours & this will be displayed using a traffic light system on the patient chart within the AKI Patientrack Assessment Pack. In addition real time Creatinine results will be fed into the AKI Patientrack Assessment Pack to highlight 'confirmed AKI' using the same traffic light system. This is demonstrated pictorially below:

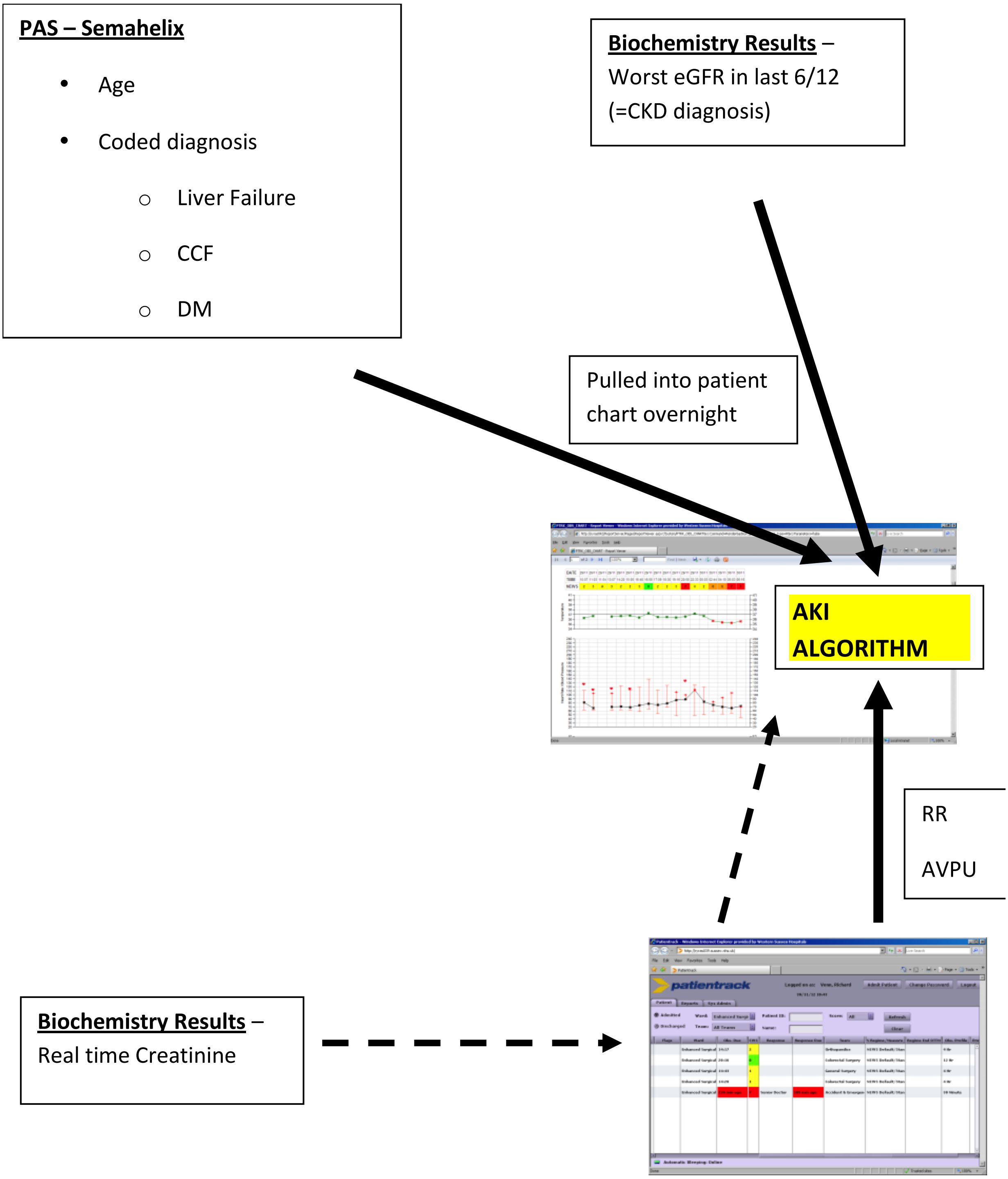

(Abbreviations: AVPU level of consciousness – Alert/Vocal/Pain/Unresponsive; CCF – congestive cardiac failure; DM – Diabetes; RR – Respiratory rate)

Results will be displayed within the patient chart as a traffic light system (No AKI, At risk, Confirmed AKI). If the patient displays ‘At risk’ of ‘Confirmed AKI’ then clicking over the traffic light key will take the clinician to a new screen displaying the Creatinine results graphically & a checklist for AKI. An automated AKI alert will be also sent at 0100 each day to the responsible Doctor with a ‘read receipt’ to acknowledge that the e-mail information has been received. All patients ‘at risk’ or ‘confirmed AKI’ will also appear on the AKI Hospital Patient List which can be accessed by the medical staff, Outreach staff & Renal team. This is displayed pictorially on the following page:

Main Patientrack frontpage

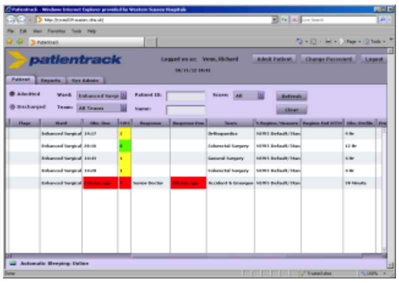

Patient Chart

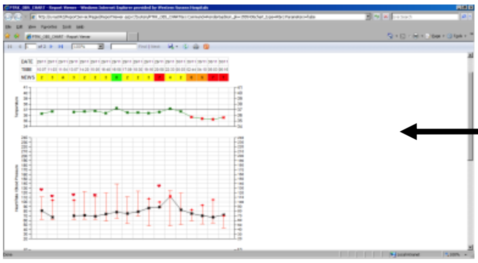

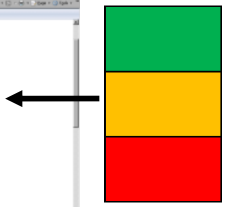

CLICK INTO

AKI Hospital Patient List

- Name
- Hospital No
- Age
- Gender
- Consultant
- Ward
- Bed number
- Most recent Creatinine
- AKI status
    - At risk
    - Confirmed

CLICK

INTO

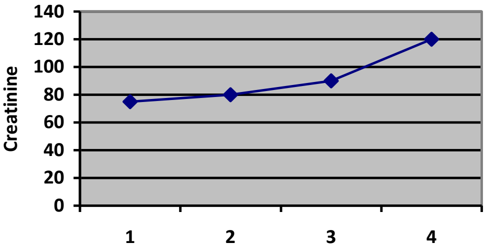

CHECKLIST

1. REVIEW FLUID BALANCE – correct hypovolaemia if appropriate
2. REVIEW DRUG CHART – stop nephrotoxins
3. EVIDENCE FOR SEPSIS? – investigate & treat urgently
4. INVESTIGATIONS
    a. Urine Dip: If 'active' seek advice
    b. Daily U&E, Creatinine
    c. Consider U/S renal tract
5. PPI if oral nutrition poor
6. Avoid contrast media unless essential (seek advice)

(Abbreviations: U&E – urea and electrolytes; U/S – ultrasound; PPI – proton pump inhibitor)

Educational Package: pre & post implementation questionnaires will be distributed to nursing & medical staff to identify any educational requirements. An educational package will be delivered to all nursing & medical staff prior to implementation consisting of e-learning, paper educational tools & small group ward-based teaching by the Outreach teams.

Post - Introduction of 'AKI Patientrack Assessment Pack' to WSHT, data collection: data will be retrieved from the Patientrack, Pathology & PAS databases relevant to the 'AKI Patientrack Assessment Pack'. This data will be transferred to an excel spreadsheet and then anonymised. This data will be stored on the WSHT server & only hospital password protected computers will be able to access this data. Anonymised data for mathematical & statistical analysis will be transferred by password protected & encrypted memory sticks or secure NHS e-mail.

3.1 STUDY OUTCOME MEASURES

Outcomes data to be collected & analysed during the interrupted time-series design:

- Incidence of AKI
- Magnitude of acute deterioration in Creatinine, eGFR
- Compliance with AKI checklist
- Mortality
- Morbidity – secondary complications including other organ failures
- LOS
- Requirement for renal replacement therapies
- Requirement for escalation to critical care

4. PARTICIPANT ENTRY

4.1 PRE-REGISTRATION EVALUATIONS

No additional tests will be performed in the study compared to if the patient was not in the study.

4.2 INCLUSION CRITERIA

Phase 1 – retrospective validation study: adult patients admitted to the Acute medical units of Chichester and Worthing and Becket ward.
Phase 2 - adult patients admitted to the Acute medical units of Chichester and Worthing and Becket ward.

4.3 EXCLUSION CRITERIA

Patients admitted to non-Patientrack wards at WSHT such as delivery suite, day surgery & paediatrics

4.4 WITHDRAWAL CRITERIA

Not applicable

5. ADVERSE EVENTS

This study involves observational analysis initially with no alteration to usual care. When the Acute Kidney Injury Alert and Patientrack Assessment Packs are introduced at the Worthing site this will constitute a care bundle of best practice advice that should not impact in any adverse way.

The Chief Investigator and the Study team will meet at least monthly to review the data and perform interim analysis of the results.

6. ASSESSMENT AND FOLLOW-UP

This is an initial in-patient study with no long-term follow-up planned outside of normal protocols.

The study ends on the patients discharge.

7. STATISTICS AND DATA ANALYSIS

Logistic regression analysis will be used to identify predictors of development of acute kidney injury within seven days of admission. The scoring system (APS) developed from a previous Logistic regression analysis will be re-evaluated. The calibration of the scoring system will be assessed by the Hosmer-Lemeshow goodness-of-fit test.[7] Discrimination will be assessed using the area under the receiver operating characteristic curve (AUC). Validation will be carried out by applying the scoring system comparing patients with AKI and those who did not develop AKI within 7 days.

Statistical analyses will be performed using SPSS v.19 with P-values below 0.05 considered as significant.

Data and all appropriate documentation will be stored for a minimum of 10 years after the completion of the study, including the follow-up period – as per MRC Good Research Practice Guidelines (2005).

8. REGULATORY ISSUES

8.1 ETHICS APPROVAL

Ethical approval will be sought and the trial will be conducted in compliance with the principles of the Declaration of Helsinki, the principles of GCP (for non-commercial trials, as defined by the Medical Research Council) and all of the applicable regulatory requirements.

8.2 CONSENT

We will be placing posters in all wards where patient data will be analysed describing the purpose of the study and the care bundles (alert packs) being trialled. The alert being

instituted initially at Worthing recommends a bundle of best practice and as such is not a research intervention that could be consented for.

8.3 CONFIDENTIALITY

Data retrieved from all these databases will be transferred to an excel spreadsheet and then anonymised. This data will be stored on the WSHT server & only hospital password protected computers will be able to access this data. Anonymised data for mathematical & statistical analysis will be transferred by password protected & encrypted memory sticks or secure nhs e-mail.

A report of the study findings will be submitted for publication in an appropriate scientific journal and/or international conference.

8.4 INDEMNITY

NHS indemnity will apply. The Chief Investigator's organisation will be the organisation on which a claim is made. Compensation provision may be made through the 'Liabilities to Third Parties' scheme of the NHS Litigation Authority.

8.5 SPONSOR

Sussex NHS Research Consortium

8.6 FUNDING

It is envisaged that an application will be made to the NIHR for an RFPB grant to help with statistical, health economic analysis and ongoing running of the research.

8.7 AUDITS AND INSPECTIONS

The Chief Investigator and the Study team will meet at least monthly to review the data and perform interim analysis of the results.

9. STUDY MANAGEMENT

Dr Lui Forni Chief investigator and his study collaborators

11. PUBLICATION POLICY

A report of the study findings will be submitted for publication in an appropriate scientific journal and/or international conference.

12. REFERENCES

1. Uchino S, Kellum JA, Bellomo R, Doig GS, Morimatsu H, Morgera S, Schetz M, Tan I, Bouman C, Macedo E, Gibney N, Tolwani A, Ronco C; Beginning and Ending Supportive Therapy for the Kidney (BEST Kidney) Investigators: Acute renal failure in critically ill patients: a multinational, multicenter study. *JAMA* 2005, 294(7):813-818

2. Hoste EA, Clermont G, Kersten A, et al. RIFLE criteria for acute kidney injury are associated with hospital mortality in critically ill patients: a cohort analysis. *Crit Care* 2006; 10: R73

3. Ostermann M, Chang RW: Correlation between the AKI classification and outcome. *Crit Care* 2008, 12(6):R144

4. Coca SG, Yusuf B, Shlipak MG, et al. Long-term risk of mortality and other adverse outcomes after acute kidney injury: a systematic review and metaanalysis. *Am J Kidney Dis* 2009; 53: 961–973

5. Stewart J, Findlay G, Smith N et al. Adding Insult to Injury. A review of the care of patients who died in hospital with a primary diagnosis of acute kidney injury (acute renal failure). National Confidential Enquiry into Patient Outcome and Death. London: NCEPOD, 2009; 1–100

6. Thomas M, Sitch A, Dowswell G. The initial development and assessment of an automatic alert warning of acute kidney injury. *Nephrol Dial Transplant* 2011; **26**: 2161-68

7. Selby NM, Crowley L, Fluck R, McIntyre CW, Monaghan J, Lawson N, Kohle N. Use of Electronic Results Reporting to Diagnose and Monitor AKI in Hospitalized Patients. *Clin J Am Soc Nephrol.* 2012; **7**: 533-40.

8. Kheterpal S, Tremper KK, Heung M, *et al*. Development and validation of an acute kidney injury risk index for patients undergoing general surgery. *Anesthesiology* 2009; **110**: 505-15

9. Schneider DF, Dobrowolsky A, Shakir IA, Sinacore JM, Mosier MJ, Gamelli RL. Predicting acute kidney injury among burn patients in the 21st century: a classification and regression tree analysis. *J Burn Care Res*. 2012; 33 :242-51.

10 Matheney ME, Miller RA, Ikizler TA, Waitman LR, Denny JC, Schildcrout JS, Dittus RS, Peterson JF. Development of Inpatient Risk Stratification Models of Acute Kidney Injury for Use in Electronic Health Records. *Med Decis Making* 2010; **30**:639-50
